# *De novo* ChIP-seq analysis

**DOI:** 10.1186/s13059-015-0756-4

**Published:** 2015-09-23

**Authors:** Xin He, A. Ercument Cicek, Yuhao Wang, Marcel H. Schulz, Hai-Son Le, Ziv Bar-Joseph

**Affiliations:** Department of Human Genetics, The University of Chicago, 920 E. 58th Street, CLSC, Chicago, IL 60637 USA; Computational Biology Department, Carnegie Mellon University, 5000 Forbes Ave, Pittsburgh, PA 15213 USA; Department of Computer Engineering, Bilkent University, Ankara, 06800 Turkey; Computer Science and Artificial Intelligence Laboratory, 32 Vassar Street, MIT, Cambridge, MA 02139 USA; Multimodal Computing and Interaction, Saarland University & Max Planck Institute for Informatics, Saarbrücken, 66123 Saarland Germany

## Abstract

Methods for the analysis of chromatin immunoprecipitation sequencing (ChIP-seq) data start by aligning the short reads to a reference genome. While often successful, they are not appropriate for cases where a reference genome is not available. Here we develop methods for *de novo* analysis of ChIP-seq data. Our methods combine *de novo* assembly with statistical tests enabling motif discovery without the use of a reference genome. We validate the performance of our method using human and mouse data. Analysis of fly data indicates that our method outperforms alignment based methods that utilize closely related species.

**Electronic supplementary material:**

The online version of this article (doi:10.1186/s13059-015-0756-4) contains supplementary material, which is available to authorized users.

## Background

Over the last few years, next generation sequencing (NGS) technologies have revolutionized our ability to study genomic data. While these techniques have initially been used to study DNA sequence data [1], they are now widely used to study additional types of dynamic and condition-specific biological data. Specifically, chromatin immunorecipitation sequencing (ChIP-Seq) has been used to identify novel motifs [2] to aid in the reconstruction of regulatory networks [3, 4] and to study the role of epigenetics in regulation [5].

The standard pipeline for analyzing these experiments starts with aligning reads to the genome to identify their origin and to correct errors. Next, peaks (regions where read abundance is enriched compared to a control) are identified and their enrichment is determined by comparing the coverage of these peaks between case and controls [6]. Several methods have been proposed to perform such peak detection and for quantifying peak enrichment [6]. While these methods differ in important aspects (including the type of distribution they assume, the method that they assign reads to genomic regions, the way in which enrichment is calculated, and so on), all current ChIP-Seq analysis methods rely on the first step mentioned above: Read alignment to the genome.

Although genome-based alignment is possible for several species, there are many cases in which alignments to the genome are either not possible or can miss important events. Assembly and annotation of complete genomes is time- and effort-consuming and, to date, less than 250 of the more than 8 million estimated Eukaryotic species have been fully sequenced at the chromosome level [7]. However, information from several related species is often required in order is to determine common processes and their evolutionary plasticity in order to understand the overarching principles of developmental biology. Consider for example the sea urchin (Stronglyocentrotus purpuratus) model. While detailed maps of developmental gene regulatory networks (GRNs) are well known for this model organism [8], comparative studies using related species including sea star and sea cucumber, which have not been fully sequenced to date, are required to resolve longstanding questions related to factors involved in sea urchin development. For example, it has long been assumed that TFs are under selection pressure and so evolve slower than other proteins [9]. Therefore change in binding targets for such factors should be predominantly cis-regulatory [10]. On the other hand, it has become increasingly appreciated that TFs can evolve biochemical differences and that these will be important to the motifs that bind to [11, 12]. Analysis of *in-vitro* binding preferences (using protein binding arrays) indicates that TFs can evolve over the evolutionary distance between sea urchin and sea star [13]. However, this analysis does not provide information about *in-vivo* binding properties, which can only be determined using ChIP-based studies. Thus, methods that can perform *de novo* analysis of ChIP-Seq data can provide important information regarding motif evolution and inform us on how binding properties of conserved TFs vary across related species.

Even when the reference genome is available, in some cases including in cancer cells, because of mutations, rearrangements, and other genomic perturbations we may not be able to fully rely on it when performing Seq experiments [14–17].

Similar to standard ChIP-Seq analysis methods, in most RNA-Seq analysis pipelines the reads are first aligned to the genome and then assembled and quantified using the genome reference. Thus, transcriptomics analysis faces similar problems when studying species for which no reference genome exists or when attempting to analyze cancer expression data [18]. Several methods for *de novo* transcriptomics analysis have been developed to address these issues [18–20]. However these methods cannot be directly applied to ChIP studies since their focus is not on peak and/or motif detection but rather on transcript assembly, and on resolving alternatively spliced transcripts.

**Fig. 1:**
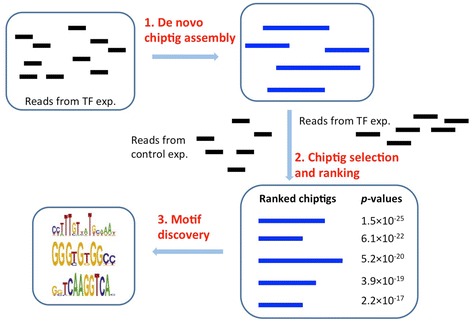
De novo ChIP-seq analysis pipeline. Top: Reads from the TF experiment are assembled using a *de novo* assembly method (SEECER or Velvet) leading to longer ChIPtigs, each of which is based on several (often hundreds or thousands) of assembled reads. Bottom right: Each of the assembled ChIPtigs is scored to determine its enrichment for experiment vs. control reads. ChIPtigs are ranked based on their enrichment scores. Bottom left: Top ranking ChIPtigs are used as input for a motif discovery method resulting in a set of motifs for the experiments

To enable experiments that study motif evolution using non-sequenced species or in cases where the reference can greatly differ from the genome being studied, we developed a new method for the analysis of *de novo* ChIP-Seq data (Fig. 1). Unlike prior methods that identify peaks following short read alignment to the genome, we first use *de novo* assembly methods originally developed for RNA-Seq to assemble longer segments that we term ChIPtigs. Using these ChIPtigs we align reads from both, the genuine and control ChIP-seq samples to these assembled ChIPtigs and use these alignments to compute an enrichment score. We next rank the ChIPtigs and perform *de novo* motif discovery on the top enriched ChIPtigs to determine binding motifs.

To test the new method we first applied it to mouse data (where we can compare it directly to methods that utilize the genome). As we show, for most TFs the *de novo* method was able to accurately detect the correct motif even without using the genome as a reference. We next analyzed ChIP-Seq data from several human cancer cell lines. This analysis further demonstrates that our *de novo* methods are able to accurately identify both the correct motifs and motifs for co-factors of the TF being studied. Finally, to simulate *de novo* analysis of a non-sequenced species, we used fly data to show that our method outperforms methods that rely on a closely related (sequenced) species when analyzing ChIP-Seq data from a non-sequenced species.

## Results

### *De novo* ChIP-seq analysis on mouse embryonic stem cell (ESC) data

The major goal of *de novo* ChIP-Seq analysis is to study species for which the genome is either not sequenced or not fully annotated or to study cases such as cancer where we expect large differences between the actual and general reference genome. Still, to test our method it is best to use a well annotated genome and dataset so that we can determine how successful the method is using ‘gold standard’ data. We have thus initially applied our method to ChIP-Seq mouse data. Using such data we can compare *de novo* motif discovery with established methods that are based on peak calling [6]. Briefly, most methods for the analysis of ChIP-Seq data start by aligning the reads to the genome and identifying ‘peak regions’ places in the DNA that are enriched in the test experiment when compared to the control. Next, these genomic regions are extracted and a motif discovery tool is used to determine the actual DNA binding motifs. We used a mouse ChIP-seq dataset that measured the binding of 15 TFs in mouse embryonic stem cells [21]. The data for each TF are composed of between 5 and 12 million reads, each of length 26 bp. We excluded three TFs from our analysis (E2F1, p300, and Suz12), since no specific motif was identified for them, even when using the reference genome. For the peak-calling method we used MACS (Zhang *et al.* 2008) [22]. We also tried CisGenome [23], but it failed to detect motives that MACS detected and so the results presented are based on MACS. For the *de novo* analysis we used both Velvet and SEECER (Methods) to generate ChIPtigs from these reads. The ChIPtigs are ranked based on their enrichment in a cases compared to controls using a statistical test. We then used our pipeline described in Methods for ranking these ChIPtigs. For motif discovery in the identified peaks or ChIPtigs, we used the tool DREME from the MEME suite. We first assessed the ChIPtigs generated by Velvet and SEECER to determine the accuracy of these methods for the *de novo* assembly task of ChIP-Seq data. Table 1 (Velvet) and Table 2 (SEECER) present some of the results of this analysis including information about the number of ChIPtigs and the fraction of ChIPtigs that could be mapped to the genome. A ChIPtig is considered to be successfully mapped, if at least 95 % of its bases can be aligned to the mouse genome. As can be seen, for most TFs, several thousand ChIPtigs are assembled and a large fraction of them can be mapped back to the reference genome (often more than 90 %), indicating that the *de novo* assembly indeed recovers many of the bound regions.

**Table 1:** ChIPtig statistics and results of motif finding in the mouse ESC dataset using Velvet

TF	No. ChIPtigs	Mapped ChIPtigs (%)	Motif rank with peak-calling	Motif rank with *de novo* pipeline	Motif rank with random ChIPtigs
c-MYC	5,159	92.9	1	1	4
CTCF	2,152	92.9	1	2	1
ESRRB	30,278	95.9	1	1	1
KLF4	1,660	90.4	1	1	1
NANOG	5,163	94.3	1	N	N
n-MYC	3,610	86.8	1	1	1
POU5F1	2,528	92.9	1	1	N
SMAD1	596	96.1	7	N	N
SOX2	2,511	92.5	1	1	N
STAT3	4,329	94.8	1	1	N
TCFCP2I1	20,566	95.7	N	N	N
ZFX	3,348	93.5	1	1	1

Three settings were evaluated for motif finding performance: peak-calling using reference genome (MACS), top 1,000 ChIPtigs from the *de novo* pipeline, and 1,000 random ChIPtigs from the same experiment assembled by Velvet. The rank of the known motif (from JASPAR) in the DREME results is shown for each TF. ‘N’ in a row means that either DREME did not find any motif, or none of the motifs found by DREME matches the known motif for the TF in that row

**Table 2:** ChIPtig statistics and results of motif finding in the mouse ESC dataset using SEECER

	No. ChIPtigs	Mapped ChIPtigs (%)	Motif rank with peak-calling	Motif rank with *de novo* pipeline	Motif rank with random ChIPtigs
c-MYC	15,987	86.5	1	1	N
CTCF	8,209	39.6	1	N	N
ESRRB	41,620	90.7	1	1	1
KLF4	10,144	73.5	1	1	N
NANOG	19,106	43.7	1	N	N
n-MYC	13,663	67.2	1	N	N
POU5F1	12,939	75.5	1	N	N
SMAD1	9,914	39.8	7	N	N
SOX2	12,797	77.7	1	1	N
STAT3	17,394	84.7	1	1	N
TCFCP2I1	31,701	89.4	N	N	N
ZFX	10,569	80.4	1	1	2

Columns are the same as in Table 1

To test if the information contained in these ChIPtigs is enough to recover the correct motifs, and if the rankings we are using help in such goal we next used our ranked ChIPtig list (top 1,000 ChIPtigs, though top 2,000 led to similar results) to search for motifs for each of the TFs and compared the results to known motifs from the TF studied and to peak-calling methods for the same data. Results for Velvet are presented in Table 1, and those of SEECER in Table 2. We also assessed the performance of motif discovery when using the standard peak-calling analysis, which relies on the reference genome. The peak-calling method was able to identify the correct motif as the top motif for 10 of the 12 TFs based on the JASPAR database (all except SMAD1 and TCFCP2I1). Using our *de novo* ChIP-Seq analysis pipeline to select the top 1,000 ranked ChIPtigs (Methods), we were able to identify the correct motif as the top motif for eight out of these 10 TFs (a 20 % drop when not using the reference genome). For an additional ninth factor (CTCF) the correct motif was ranked second in our analysis. In contrast, when only using a random subset of the assembled ChIPtigs (that is, using 1,000 ChIPtigs selected at random from those assembled by Velvet from the ChIP-Seq experiment reads), only four TFs had the correct top-scoring motif (a drop of 60 % compared to baseline).

We have also compared the overlap between the detected ChIPtigs and the peaks detected by the peak-calling analysis. We have mapped the top 2,000 ChIPtigs we have obtained from each analyses to the genome. Varying the cutoff for the percentage of the ChIPtig mapped to the genome, we have obtained the ratio of the ChIPtigs overlapping with the peaks. Results show that even using 80 % as the mapping cutoff, 50 % of the peaks on average are found by using Velvet (53 %) and more than 40 % by using SEECER. Please see Additional file 1: Tables S1 and S2 for detailed results.

In summary, our analysis demonstrates that a significant fraction of the ChIPtigs assembled from short-reads is likely regions bound by TFs (as even randomly chosen ChIPtigs enable motif discovery in some cases), and that our ranking function (Methods) can accurately identify bound ChIPtigs, which improves downstream analysis. In terms of motif discovery, our *de novo* pipeline performs only slightly worse than the peak-calling method, which has the benefit of reference genomes.

### Analyzing human ChIP-Seq data

The mouse data described above provide a way to test *de novo* ChIP-Seq analysis in cases where the motif and reference genome are known and so genome based peak-calling methods should be the optimal strategy. It is thus not surprising that *de novo*-based methods are not doing as well as peak-calling methods. Still, the results above indicate that *de novo* motif finding can be successful in several cases indicating that it is a viable option for species without an available reference sequence. To further test the ability of *de novo*-based analysis to accurately identify DNA binding motifs we next asked how well it could perform on human cancer data. While reference genome sequence information is still available for human cancer data, several cancer cell lines display significant genomic alterations when compared to normal samples of the same tissue from the same individual indicating that the advantages of using genome based peak-calling methods may be diminished for such data. We have thus compared the analysis cancer ChIP-Seq data using our *de novo* pipeline to the analysis of the genome-based peak-calling methods SeqPeak and MACS.

**Fig. 2:**
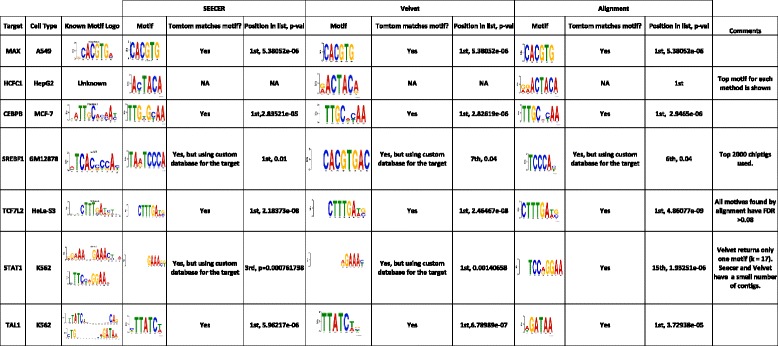
Motif discovery results for the human validation data. The table presents the results obtained for each of the TFs (rows) using the *de novo* assembly pipeline with SEECER and Velvet and the results for the peak-calling method MACS. For each TF we present the known motif (if it exists in the database). For each method we show: (1) the predicted motif that best matches the known motif; (2) whether it matches the known motif in the JASPAR database; and (3) the motif rank in the DREME results for that method and the TOMTOM *P* value for the match with the known motif. We also include experiment specific comments in the last column

We selected seven TFs from several different cancer cell lines for this analysis (Fig. 2). Read data for all factors were downloaded from the ENCODE project repository [24]. For each of the TFs we studied we selected a specific cancer type (corresponding immortalized cell line) and have downloaded both the case and control experiment for that factor. Six of the seven TFs have a known motif while HCFC1 had no known annotated motif in the Jaspar database [25]. For each of the seven ChIP-Seq datasets, we performed peak calling using SeqPeak and MACS followed by motif discovery using DREME [26]. For the *de novo* pipeline we have used Velvet and SEECER (as described in Methods) to identify enriched ChIPtigs followed by DREME to perform the motif discovery. We have next used TOMTOM [27] to compare the top motifs for each TF/method with motifs in the Jaspar database. The results for the *de novo* methods and the MACS peak calling are presented in Fig. 2 and results for SeqPeak are presented in Additional file 1: Figure S1. Since MACS clearly outperformed SeqPeak we only focus on the MACS results in the discussion below. Overall, as can be seen in Fig. 2, *de novo*-based methods performed very similarly to sequence-based methods (in some cases even improving upon them, see below), a significant improvement over the comparison presented in Table 1, which analyzed data from normal tissues. For five of the seven TFs, both methods were able to identify to correct motif as the top motif, though they slightly differed in how well they recovered the motif based on the TOMTOM *P* value match statistics. Interestingly, even though the *de novo* methods did not use the reference genome, the *P* value they obtained for one of these factors (TAL1) was better than the *P* value obtained by the peak-calling method. A possible explanation for this result is that our method finds a binding motif of length 7 which leads to higher significance than the reference-based motif which is of length 6. The motif found is TAL1::GATA1 motif, to which GATA1 and TAL1 binds cooperatively. TAL1 binds to the CTG part of the motif shown in Fig. 2. As for the other two TFs, for SREBF1, SEECER was able to identify the correct motif as a top hit, and MACS identified it as a lower hit and was able to recover a portion of the known motif.

### Analysis of co-factors

In addition to identifying motifs for the factors being studied, ChIP-Seq datasets can often be used to identify motifs for co-factors of the TF being analyzed (Bailey *et al.* 2011) [26]. Thus, the presence of motifs for known co-factors of a TF can serve as an indication that the read analysis method (either *de novo* or alignment) is accurately capturing the biological information in the dataset. We have thus intersected the TOMTOM TF matches for the top 10 motifs identified for these eight factors with interaction data from the human protein reference database (HPRD) [28]. For each TF we determined whether any of its top 10 motifs match a motif for a known co-factor. The results are presented in Table 3 (see supporting website for complete results). Again, the results indicate that for the human validation data *de novo* and peak-calling methods are comparable, mostly identifying a similar set of correct co-factors. The only exceptions here are CEBPB (SP1 and EGR1) and TAL1 (SP1) where the *de novo* analyses of SEECER and Velvet correspondingly were able to identify a motif for a co-factor that MACS did not identify. MACS, on the other hand, identified a motif for co-factor of CEBPB (FOXO1), which the *de novo* methods did not find.

**Table 3:** Co-factors identified in the top 10 motifs predicted by each method for the human validation dataset

	SEECER	Velvet	Alignment
MAX	*MYC, MYCN*	*MYC, MYCN*	*MYC, MYCN*
HCFC1		*GABPA, SP1*	*GABPA, SP1*
CEBPB	*CEBPA*	*CEBPA, SP1, EGR1*	*FOX01, CEBPA*
SREBF1		*SP1*	*SP1*
TCF7L2			
STAT1			
TAL1	*SP1, GATA3*	*TCF3*	*GATA3, TCF3*

Proteins that are found to be interacting with the target and whose motifs are predicted in the top 10 by each method are shown

### Effect of number of ChIPtigs used on performance

In order to test the effect of varying number of ChIPtigs used in the *de novo* pipeline, we tested Velvet’s performance on the human validation dataset using top 1,000 ChIPtigs, top 2,000 ChIPtigs, and using all available ChIPtigs. We checked if the ranking of the correct motif changed with respect to varying number of ChIPtigs. We excluded HCFC1 for this analysis, as there is no known motif for it and excluded STAT1 as Velvet only returns 415 ChIPtigs. Only the highest ranked motif was considered when there are more than one available known motifs. As shown in Additional file 1: Table S3, the ranking of the top discovered motif did not change as the number of ChIPtigs varied for MAX, CEBPB, and TAL1. For SREBF1, we could not match the correct motif using top 1,000 ChIPtigs, but using top 2,000 and using all returned the correct motif at the seventh spot. Finally, for TCF7L2, increasing the number of ChIPtigs has deteriorated the results.

### Simulating a *de novo* ChipSeq motif discovery using fly species data

A key goal of our pipeline is to provide a motif discovery tool to researchers working on organisms without a sequenced genome. To test the usefulness of our approach we have simulated such a case with two fly species: D. Melanogaster and D. Pseudoobscura.

**Fig. 3:**
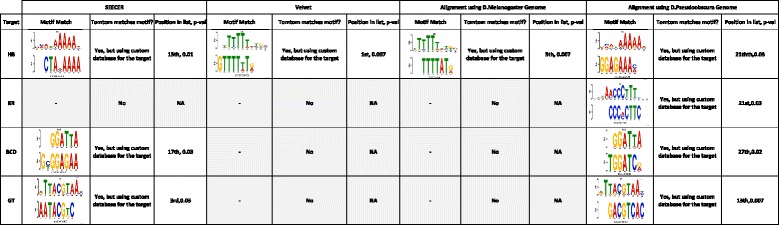
Motif discovery results for the fly data. The table presents the results obtained for each of the TFs (rows) using the genome based motif discovery using D. Melanogaster genome and using D. Pseudoobscura genomes and *de novo* assembly pipeline with SEECER and Velvet. For each TF we present the known motif (if it exists in the database). For each method we show: (1) the predicted motif and how the predicted motif matches (if any); (2) whether it matches the known motif in the JASPAR database; and (3) the motif rank in the DREME results for that method and the TOMTOM *P* value for the match with the known motif

While both have been sequenced, if we do not use the D. pseudoobscura in the analysis (to simulate a case where a species has not been sequenced) the closest genome we could use for an alignment based peak-calling method is D. melanogaster. We obtained four chipseq datasets for D. pseudoobscura for the following transcription factors: BCD, GT, HB, and KR [29]. We performed motif discovery using: (1) standard peak-calling using the D. pseudoobscura genome (as a sanity check); (2) standard peak-calling using the D. melanogaster genome; (3) *de novo* analysis using Velvet; and (4) *de novo* analysis using SEECER. Standard peak-calling was performed using MACS. Results are shown in Fig. 3. As expected, MACS was able to detect the known motifs using D. pseudoobscura genome for all transcription factors. However, it could not detect the correct motif for three of the four factors when using the D. melanogaster genome. In contrast, using the *de novo* analysis pipeline with SEECER we were able to identify the correct motif for three out of four TFs. Velvet identified the correct motif for only one factor, the same as the only one identified by the alignment based method (HB). However, the correct motif for HB was ranked higher by Velvet (first) than the correct motif found by the alignment method (which only ranked fifth). Thus, both Velvet and SEECER improve upon alignments to closely related species when performing *de novo* analysis.

## Conclusions

To date, the analysis of ChIP-Seq data has relied on peak calling based on the alignment to a reference genome. While several of the methods developed for this task have been highly successful, the requirements for a reference genome prevented the use of this technology for non-sequenced species. In addition, reliance on genome alignment may be problematic in cases where the genome being investigated is very different from the reference, such as cancer cells [17].

Here we presented a new pipeline for the *de novo* analysis of ChIP-Seq data. Unlike prior methods we do not start by aligning short reads to the genome. Instead, we first assemble short reads into longer ChIPtigs by modifying methods originally developed for *de novo* RNA-Seq analysis [19]. Next, we identify ChIPtigs that are enriched for test reads compared to control reads and rank them using a statistical test. The ranked list of ChIPtigs is then analyzed to identify motifs that are likely the target of the TF being studied. Finally, the highly ranked ChIPtigs that contain a motif of interest (which are much longer and more accurate than the individual reads) can in some cases be aligned to partially assembled genomes or to known genomes of closely related species to determine potential targets of the TF being investigated. Combined, this *de novo* analysis pipeline provides a solution that spans both motif discovery and the ability to determine the TF function and regulatory subnetwork based on the identified targets (in the same or related species).

We first tested our method on known motifs from normal mouse tissues. For such data we expect the peak-calling method to be optimal and so it can serve as a test set for expected accuracy reduction when applying *de novo* methods to non-sequenced species. As we show, the *de novo* pipeline performed very well enabling us to correctly identify eight motifs as top hits, compared to 10 motifs identified with current peak-calling methods. Note that this comparison is extremely challenging for our *de novo* analysis pipeline since the original data was based on very short reads (26 bp) which are thus harder to assemble in a *de novo* manner. We expect that the results would be even better for longer reads.

We next tested our method using human cancer data. While cancer genomes are still quite similar to the human reference genome they often suffer from a high mutation rate [17]. These mutations may make it harder to correctly align the short reads to the reference genome and in some cases can lead to inability to identify such alignments or to find regions that are enriched in the case vs. control studies (peaks). In contrast, *de novo* analysis of such read data, which does not rely on the reference genome, may still be able to identify enriched ChIPtigs even if the reads used to generate the ChIPtigs differ from their original reference due to mutations and rearrangements. Indeed, when analyzing data from cancer TF studies we found that *de novo* ChIP-Seq analysis performs as well as peak-calling methods, and in some cases it even slightly improves over alignment based methods. For four of the seven TFs the motifs identified using the *de novo* methods had a better *P* value match to the correct motif when compared to the motifs identified using the peak-calling methods.

While ChIP-Seq data for non-sequenced species are not yet available (at least to some extent since no method currently exists to analyze it), we can simulate such cases when data are available for a number of closely related species. We have thus analyzed fly data by assuming that one fly species does not have a sequenced genome, while its close evolutionary relatives have. We show that while the genome of the evolutionary relative fails to be useful for identifying the correct motif for four transcription factors, our *de novo* method was able to recover the correct motif in three of those transcription factors. While the ranking of the correct motif is low for these factors, we observe a similar low rank for the correct motif when using a genome-based alignment with the true genome. Note that the known motif is based on D. melanogaster and it could be the case that in D. pseudoobscura these proteins may bind to other motifs. In fact, Paris *et al.* state that the number of peaks detected for D. pseudoobscura is lower [29], which indicates that there might be different binding specificities between these fly species.

In our comparison of *de novo* and alignment-based methods, we tested aligning the reads to the genome of a related species. While longer segments (for example, the ChIPtigs we generate) may lead to better alignments, it is unlikely that they would lead to better motifs, because read alignment not leading to the correct motifs indicate that there is a divergence between the two species at the binding sites.

The ability to perform *de novo*-based analysis of ChIP-Seq data opens the door to several possibilities. These include: (1) comparison of binding motifs for mutated versus wild-type transcription factors; and (2) motif evolution analysis in developmental studies, for species that have not been fully sequenced. It also enables researchers to analyze data from studies in which we may expect the genome being analyzed to diverge from the reference genome for that species. We provide a fully implemented pipeline for such *de novo* analysis (using either velvet or SEECER) on the supporting website. We hope that our pipeline will serve as a complementary procedure to genome based alignment methods when performing ChIP-Seq studies.

## Methods

### A *de novo* ChIP-seq analysis pipeline

We developed a computational pipeline to extract the TF binding motifs from ChIP-seq data, assuming no reference genome is available. The input data are the short reads from a ChIP-seq experiment of the TF being studied, and from a control experiment where non-specific antibody or input DNA is used. The pipeline has three main steps (Fig. 1). First, we perform *de novo* ChIPtig assembly on the reads obtained in the ChIP-seq experiment of the TF – such ChIPtigs would represent putative regions bound by the TF. In the second step, reads in both TF and control experiments are mapped to these ChIPtigs, and the ChIPtigs are then selected and ranked by their enrichment for the TF vs. control experiments. Finally, a motif finding program is used to identify motifs in the most enriched ChIPtigs using a ranking that is based on the statistics computed in step 2. The details of each step are described below. Please also see Additional file 1: Text 1 for instructions on how to use the pipeline and the supplementary website for the implementation.

### *De novo* ChIPtig assembly

Since no reference is available, the first step is focused only on the actual ‘case’ experiments (binding of the real TF). Note that unlike other datasets for which *de novo* assembly is used (most notably RNA-Seq [30]) here the assembly task is less challenging. Specifically, while in RNA-Seq we may need to handle alternatively spliced regions leading to branch points in the assembly, ChIP-Seq data are mostly retrieved from continuous DNA sequences and so assembly can be done more accurately. We tested two methods for such assembly: Velvet and SEECER. Velvet is a popular *de novo* genome assembly tool for short read sequencing data based on De Bruijn graphs [31]. In the de Bruijn graph, reads are encoded as paths in the graph spelling the k-mers they contain. A vertex (node) represents a k-mer, and an edge linking two nodes represents an overlap of k-1 nucleotides between the nodes’ sequences. Following iterative error removal steps, which remove short nodes and redundant paths, the remaining linear paths in this graph are connected by Velvet to form ChIPtigs. Velvet was shown to construct DNA sequences efficiently from de Bruijn graphs, while eliminating errors and resolving repeats at the same time. SEECER, is a Hidden Markov model (HMM) based *de novo* assembly and error correction method [19]. SEECER learns HMMs (one for each ChIPtig) which are used to assign reads to ChIPtigs and correct errors within the short reads. The resulting ChIPtigs from each HMM represent the assembled, bound, DNA region. SEECER error corrected ChIPtigs were shown to improve error correction when compared to other error correction methods leading to better assembly of the short reads and making it an attractive method for *de novo* analysis [19].

### Adjusting SEECER for *de novo* ChIP-Seq analysis

For SEECER, we made some changes at the ChIPtig extension step. The general idea of SEECER is: first, it uses a set of highly similar reads to construct an initial ChIPtig and uses the alignment of these reads to generate an initial ChIPtig HMM (disagreements for specific columns in the aligned reads are encoded as probabilities, either emission or transition, in the HMM). Next the HMM is extended by retrieving reads that are partially aligned to the end points of the current ChIPtig HMM (the unaligned bases are used to learn the new columns of the HMM).

We use entropy to determine a stopping criteria for the HMM learning. Entropy is a probabilistic statistic which captures the uncertainty in the discrete distribution of emissions. Positions with high entropy (here we use a default max entropy = 0.6) indicate that the current aligned reads may not come from the same underlying genomic location. However, unlike RNA-Seq analysis, where such locations may indicate that we have reached an alternative splicing point (and so some reads come from one splice variant whereas the others come from another, but the transcript is not fully assembled) for ChIP-Seq we expect a continuous ChIPtig for each binding location. Thus, unlike for the original SEECER implementation when reaching a high entropy position ChIPtig extension is terminated and the resulting HMM is used as a ChIPtig (fully assembled ChIPtig for a specific binding event). Another difference between RNA-Seq and ChIP-Seq analysis using SEECER is on handling ChIPtig extension. In the RNA-Seq version of SEECER, we fix all parameters learned for the HMM prior to such extension and so the added reads that partially overlap the endpoints of the HMM do not impact the emission and transition parameters for these positions (they are only used in the extended positions). Such a block based online learning approach, which follows [32], improves runtime but can result in lower accuracy, especially if read coverage for a specific binding event is not very high.

We thus used a variant online learning methods for HMMs [33, 34] to improve the accuracy of the reconstructed ChIPtigs. Let a_i,j_ = p(q_t_ = j| q_t-1_ = i) be the transition probability, where qt is the state at time t. Let b_j_(o) = p(o|q = j) be the emission probability. To learn a HMM we need to determine the expected counts for states and transitioning between states. Let γ_t_ (i) = p(q_t_ = i|O,λ) be the expected count for states at time t (where λ represents the HMM parameters) and let ε_t_ (i,j) = p(q_t_ = i,q_(t+1)_ = j│O,λ) be the expected transition counts for that time. HMM learning involves iterative steps (based on an EM algorithm) in which either the counts are updated using the parameters or the parameters are updated using the new counts. Online learning involves sequential updates of these counts and parameters for each new observation. Since we are learning tens of thousands of such HMM models, we cannot use the full Forward-Backward algorithm for each additional read we add when extending the HMM to learn a new model. Instead, we set:

ε_t_^(r+1)^ (i,j) = αε_t_^r^ (i,j) + (1-α)ε_t_^R^(i,j) where ε_t_^r^ (i,j) is the expected transition count for state t after seeing r reads, and ε_t_^R^(i,j) is the expected value for the new read R using the current values for a and b (or for a new set of reads using the same parameters). The state counts are updated in a similar way. The discount factor α goes down as a function of r and helps guarantee that no specific read leads to large deviations from the current model.

SEECER also has a key parameter, k, the length of the kmer used to define the initial set of highly similar reads (all reads sharing the same k-mer will be included in the initial set). For our mouse analysis, because the reads we use are short (26 bp), we used k = 17 which is lower than the read length. We have used k = 19 for the cancer analysis (read length ranges between 30 bp and 50 bp), except for STAT1 analysis, for which we used k =17, as the read length was 27 bp (for both case and control).

### Using velvet with ChIP-Seq data

Velvet is a popular *de novo* genome assembly tool for short read sequencing data based on de Bruijn graphs [30]. To date, Velvet has been primarily used for *de novo* analysis of transcriptomics data [19]. Here we discuss how we extend Velvet for our *de novo* ChIP-seq analysis pipeline. In the de Bruijn graph, reads are encoded as paths in the graph spelling the k-mers they contain. Let *G* = (*V,E*) be a de Bruijn graph, where each node *n* ∈ *V* corresponds to a k-mer s ∈ ∑^*k*^ over the nucleotide alphabet *Σ* = {*A*, *C*, *G*, *T*}. An edge *e* ∈ *Ε* connects two nodes *n*_*1*_ and *n*_*2*_, iff *s*_*1*_ and *s*_*2*_ overlap by exactly *k-1* nucleotides. When using Velvet for ChIP-Seq analysis we also associate each node *n* with node *n’* that corresponds to the reverse complement k-mer of *n* to guarantee that ChIPtigs can be recovered from DNA reads that come from both strands of the genome. This is specifically important for constructing ChIPtigs, as bound DNA fragments in ChIP-seq experiments show anti correlated abundance of reads on the Watson and Crick strand of the DNA [22]. When using Velvet we first extract the set of k-mer sequences from each read to construct the set of nodes *V*. Edges are generated accordingly based on overlap between k-mers. Second, all linearly connected subgraphs, that is, nodes with one incoming and one outgoing edge are merged into the same node. Third, the following error removal steps are performed on *G* iteratively: a short chain of nodes (cumulative length <2k) that is disconnected on one end is removed and bubbles induced by highly similar sequences are collapsed. Bubbles are found by performing a Dijkstra-like breadth-first search (named Tour Bus): starting from an arbitrary node alternative paths are discovered, their corresponding sequences are extracted, aligned against each other and collapsed if they are within a sequence similarity threshold. Afterwards, low coverage nodes are removed from the graph. These steps prevent false positive ChIPtigs resulting from sequencing errors to be reported by the algorithm. After the error removal step all linear subgraphs are again merged and the assembler outputs the corresponding sequences of all nodes with length greater than a predefined threshold.

### ChIPtig selection and ranking

From the output of the ChIPtigs assembly step, we first discard all ChIPtigs that are shorter than 50 bp or longer than 500 bp as we do not expect bound regions to be much larger than 500 bp. All ChIPtigs are then scored by their enrichment in the TF experiment vs. control. Specifically, for a given ChIPtig, C, let *x*_1_ and *x*_0_ be the number of reads mapped to this ChIPtig from the case experiment (*x*_1_) and the control (*x*_0_). Denote by r the ratio between the number of reads in the TF experiment and the number of reads obtained from the control experiment (that is, r = *x*_1_ / *x*_0_). If C is not enriched in the TF experiment, then any random chosen read among (*x*_1_ + *x*_0_) reads will have a probability *r/(r + 1)* (or equivalently *x*_1_/(*x*_0_ + *x*_1_)) to occur in the TF experiment. We next use a binomial distribution to determine the enrichment of reads mapped to C in the case experiment: we ask what is the probability of observing x1 or more successes in (*x*_1_ + *x*_0_) trials with the probability of each success per trial equal to *r/(r + 1).* The *P* value is thus defined as:

1 $$ p={\displaystyle \sum_{k={x}_1}^{x_1+{x}_0}\left(\begin{array}{c}\hfill {x}_1+{x}_0\hfill \\ {}\hfill k\hfill \end{array}\right)}{\left(\frac{r}{r+1}\right)}^k{\left(\frac{1}{r+1}\right)}^{x_1+{x}_0-k} $$

After scoring each ChIPtig, the total set of ChIPtigs are ranked by their *P* values.

### Motif discovery

We use DREME for discovering motifs in the top M chiptigs returned by the previous step (where M is a parameter of the method). DREME is a discriminative motif finding tool that searches for short motifs (represented by k-mers of length up to 8, allowing degenerate symbols) that are overrepresented in the input sequences, as compared with the background sequences [26]. DREME uses Fisher’s exact test to determine the significance of the found motifs. The motifs that are found by DREME can then be compared to the motifs identified by *in-vitro* methods (for example, from protein binding microarray experiments [35]) or to known motifs from several different databases. For this comparison we relied on the Jaspar database [25] and compared motifs using the TOMTOM tool [27]. TOMTOM computes the significance of a motif in a database matching the query motif using E-values, the expected number of times that the query would match a target in a randomized database of the same size. Whenever TOMTOM could not detect any motives, we reran TOMTOM using a custom database which only includes the known motif for the target TF, to see if we can detect the motif when other known motifs in the database are eliminated.

### Accession numbers

#### Human validation analyses

MAX: ENCFF000VPU (Control: ENCFF002ECM)

HepG2: ENCFF002EDN (Control: ENCFF000PPC)

CEBPB: ENCFF000QLL (Control: ENCFF001HUM)

SREBF1: ENCFF000WDW (Control: ENCFF002ECR)

TCF7L2: ENCFF000XML (Control: ENCFF000XOO)

STAT1: ENCFF000YOW (Control: ENCFF000YOV)

TAL1: ENCFF000ZBR (Control: ENCFF000YOV)

#### Fly analyses

Experiment Series: GSE50773

Control: GSM1228798

BCD: GSM1228856

GT: GSM1228857

HB: GSM1228858

KR: GSM1228859

## Additional file

Additional file 1:
**Supplementary information for** ***de novo*** **ChIP-Seq analysis.** (PDF 230 kb)
