# Supplementary material for: De novo ChIP-seq analysis
Source: Genome Biol. 2015 Sep 23;16(1):205. doi: 10.1186/s13059-015-0756-4 (PMC4579611; doi:10.1186/s13059-015-0756-4)
Supplement: Additional file 1: — Supplementary information for de novo ChIP-Seq analysis. (PDF 230 kb) [file 13059_2015_756_MOESM1_ESM.pdf]

Supplementary Information for De Novo ChIP-Seq analysis

Xin He†, A. Ercument Cicek†, Yuhao Wang†, Marcel Schulz, Hai-Son Le, Ziv Bar-Joseph*

*Corresponding author
† These authors have contributed equally

Figure 1. Motif discovery results obtained using the SeqPeak (Ji et al. 2008). For each motif (1) known motif (if any), (2) motif found by SeqPeak, (3) if that motif is matched to the database and (4) rank and corresponding p-value of the motif are shown.

Target	Cell Type	Known Motif Logo	Motif	Tomtom matches motif?	Position in list, p-val
MAX	A549	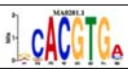	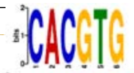	Yes	1st, 5.38052e-06
HCFC1	HepG2	Unknown	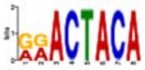	NA	1st
CEBPB	MCF-7	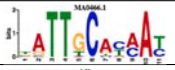	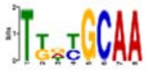	Yes	1st, 2.30438e-08
SREBF1	GM12878	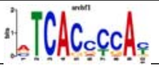	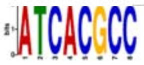	Yes, but using custom database for the target	14th, 0.0004
TCF7L2	HeLa-S3	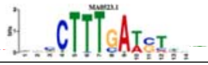	None	NA	NA
STAT1	K562	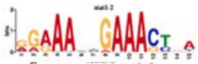 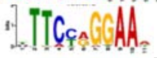	None	NA	NA
TAL1	K562	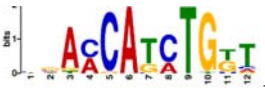	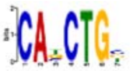	Yes	2nd, 1.89048e-05

Supporting Table 1 Ratio of the chiptigs found by SEECER (k=17) that are overlapping with the peaks detected by peak-calling using the genome. Table is constricted by picking the top 2000 chiptigs from SEECER's results and then mapped to the genome. Columns show the percentage cutoff for the overlap between the chiptig and the peak. Result for each TF analyzed in mouse is shown.

	40%	50%	60%	70%	80%	90%
c-Myc	0.402	0.4015	0.4	0.3905	0.382	0.34
CTCF	0.1525	0.141	0.1225	0.096	0.0575	0.031
E2f1	0.878	0.87	0.86	0.8435	0.8085	0.715
Esrrb	0.9405	0.9365	0.9155	0.886	0.8515	0.7575
Klf4	0.6375	0.6355	0.6265	0.595	0.5395	0.4065
Nanog	0.2465	0.2355	0.218	0.185	0.152	0.112
n-Myc	0.149	0.139	0.121	0.1045	0.0955	0.0845
p300	0.053	0.0525	0.051	0.0485	0.047	0.04
Pou5f1	0.5265	0.5235	0.515	0.498	0.4595	0.3495
Smad1	0.2235	0.2	0.1625	0.0945	0.0285	0.006
Sox2	0.5495	0.547	0.541	0.523	0.4875	0.399
STAT3	0.469	0.466	0.4615	0.453	0.4305	0.364
Suz12	0.161	0.1605	0.16	0.159	0.1535	0.14
Tcfcp2I1	0.974	0.9735	0.964	0.9495	0.932	0.888
Zfx	0.736	0.7315	0.722	0.706	0.657	0.5475
**Average**	**0.473**	**0.467**	**0.456**	**0.435**	**0.405**	**0.345**

Supporting Table 2 Ratio of the chiptigs found by Velvet (k=17) that are overlapping with the peaks detected by peak-calling using the genome. Table is constricted by picking the top 2000 chiptigs from Velvet 's results and then mapped to the genome. Columns show the percentage cutoff for the overlap between the chiptig and the peak. Result for each TF analyzed in mouse is shown.

	40%	50%	60%	70%	80%	90%
c-Myc	0.5425	0.5415	0.541	0.5365	0.526	0.4825
CTCF	0.9425	0.9425	0.937	0.913	0.842	0.694
E2f1	0.959	0.959	0.9575	0.9545	0.9505	0.9395
Esrrb	0.9775	0.977	0.974	0.9695	0.965	0.948
Klf4	0.266	0.2655	0.2635	0.257	0.247	0.214
Nanog	0.9755	0.9755	0.9735	0.962	0.9435	0.8445
n-Myc	0.622	0.6215	0.6195	0.6145	0.5955	0.544
p300	0.0365	0.0365	0.036	0.0335	0.0285	0.023
Pou5f1	0.2475	0.247	0.2435	0.2405	0.2305	0.2065
Smad1	0.003	0.003	0.003	0.003	0.003	0.003
Sox2	0.3355	0.3345	0.331	0.324	0.3065	0.265
STAT3	0.2535	0.2535	0.2525	0.251	0.244	0.2325
Suz12	0.262	0.262	0.2595	0.257	0.25	0.2335
Tcfcp2I1	0.9865	0.9865	0.985	0.9825	0.9785	0.964
Zfx	0.857	0.8545	0.85	0.838	0.8135	0.7275
**Average**	**0.5511**	**0.550666667**	**0.548433333**	**0.542433333**	**0.528266667**	**0.4881**

Supporting Table 3. The ranking of the correct motif (top ranked one if there are more than one) varying the number of chiptigs used with Velvet. Results for cancer related TFs whose motif is known are used. Velvet returns only 415 chiptigs for STAT1 analysis, which uses k =17 as kmer length. Thus, not top 1000 or top 2000 results are provided. The rest of the analyses use k = 19.

	Top 1000 ChIPtigs	Top 2000 ChIPtigs	All ChIPtigs
MAX	1st	1st	1st
CEBPB	1st	1st	1st
SREBF1	Not Found	7th	7th
TCF7L2	1st	2nd	5th
TAL1	1st	1st	1st

Supporting Text 1

Installation Guide

The pipeline requires 5 third party software tools: CD-HIT, meme suite, SEECER, razers3 and velvet These software are already have compiled version in the *third-party* folder, in the software package provided (See supplementary web-site). If user wants to manually compile these software, the following commands should be used.

1. Compile SEECER
Requirements: SeqAn, GNU Scientific Library
cd SEECER-0.2/
cd jellyfish-1.1.4/
./configure
make
cd ..
cd SEECER/
./configure
make

2. Compile Velvet
cd velvet
make

3. Compile meme
tar zxf meme_4.9.0_4.tar.gz
cd meme_4.9.0/
./configure --prefix=${software_home_directory}/third-party/meme --with-url="http://meme.nbcr.net/meme"
make
make test
make install
(Here ${software_home_directory} is the home directory of software package)

4. Compile CD-HIT
cd cd-hit-v4.6.1/
make

5. Compile RazerS3

```
cd seqan-trunk/
mkdir build/Debug
cd build/Debug
cmake ../.. -DCMAKE_BUILD_TYPE=Debug
make test_basic
./core/tests/basic/test_basic
```

How to use this platform

To run this pipeline, after it has been installed using the user guide above (or using the already compiled version). User should first move to the script directory and run the program as: ./pipeline.sh <case read file> <control read file> <output directory> <kmer length> <list of motif databases (separated with ":")> <"1" to use SEECER, "0" for velvet>. Before running the program, user should first change the config.sh file to change ROOTDIR into home directory of this software package.

Parameters Used for the Algorithms

Macs: Used the default parameters. We performed some tests enabling PeakSplitter in the pipeline. This was done using the following parameters: *"--bdg --call-subpeaks*".

Velvet: It was run via running two different programs, namely Velveth and Velvetg. Velveth was input with the k-length 19 for all transcription factors except STAT1, for which we inputed 17. Only other parameter supplied was *"-short*". Velvetg was input with the parameter *"-cov_cutoff 1.5".*

SEECER: It was input the following arguments: *"-s 1 -k 19 -p "--failureTh=3 --eDelta 0.05 --entropy=1.0 --maxCorrections 3 --no-reuse-reads"*

RAZERS: It was input the following arguments: *"-m 5 -tc 8 -i 90".*

CD-HIT: used with the default parameters.

DREME: It was input the following parameters. *"-eps –e 0.05 –v 5 -oc"*

TOMTOM: Tomtom was called using the following parameters: *"-eps –verbosity 1 -min-overlap 5 –dist pearson –evalue –thresh 0.05 –oc"*

SeqPeak: It was called using the following parameters: *"-minlen 3 -b 6 -w 12 -dat 1"*

References

Ji H, Jiang H, Ma W, Johnson DS, Myers RM, Wong WH et al, *An integrated software system for analyzing ChIP-chip and ChIP-seq data*. Nature Biotechnology, 2008. **26**: 1293-1300.
